# A Novel Xeno-Free Method to Isolate Human Endometrial Mesenchymal Stromal Cells (E-MSCs) in Good Manufacturing Practice (GMP) Conditions

**DOI:** 10.3390/ijms23041931

**Published:** 2022-02-09

**Authors:** Stefano Canosa, Katia Mareschi, Elena Marini, Andrea Roberto Carosso, Sara Castiglia, Deborah Rustichelli, Ivana Ferrero, Gianluca Gennarelli, Benedetta Bussolati, Alberto Nocifora, Valentina Asnaghi, Massimiliano Bergallo, Ciro Isidoro, Chiara Benedetto, Alberto Revelli, Franca Fagioli

**Affiliations:** 1 Gynecology and Obstetrics 1U, Physiopathology of Reproduction and IVF Unit, S. Anna Hospital, Department of Surgical Sciences, University of Torino, 10126 Torino, Italy; s.canosa88@gmail.com (S.C.); andrea88.carosso@gmail.com (A.R.C.); gennarelligl@gmail.com (G.G.); chiara.benedetto@unito.it (C.B.); alberto.revelli@unito.it (A.R.); 2 Department of Public Health and Paediatrics, University of Torino, 10126 Torino, Italy; elena.marini@edu.unito.it (E.M.); massimiliano.bergallo@unito.it (M.B.); franca.fagioli@unito.it (F.F.); 3 Stem Cell Transplantation and Cellular Therapy Laboratory, Paediatric Onco-Haematology Division, Regina Margherita Children’s Hospital, City of Health and Science of Torino, 10126 Torino, Italy; scastiglia@cittadellasalute.to.it (S.C.); drustichelli@cittadellasalute.to.it (D.R.); iferrero@cittadellasalute.to.it (I.F.); 4 Molecular Biotechnology Centre, Department of Molecular Biotechnology and Health Sciences, University of Torino, 10126 Torino, Italy; benedetta.bussolati@unito.it; 5 Department of Oncology, Pathology Unit, University of Torino, 10126 Torino, Italy; alberto.nocifora@gmail.com; 6 Department of Laboratory Medicine, Medical Genetics Division, City of Health and Science of Torino, 10124 Torino, Italy; valentina@asnaghi.net; 7 Paediatric Laboratory Regina Margherita Children’s Hospital, City of Health and Science of Torino, 10126 Torino, Italy; 8 Department of Health Sciences, University of Piemonte Orientale, 13100 Novara, Italy; ciro.isidoro@med.uniupo.it

**Keywords:** endometrial mesenchymal stromal cells, Good Manufacturing Practice (GMP), infertility, Asherman’s syndrome, endometrial thickness, human platelet lysate (HPL), endometrial sampling

## Abstract

The cyclic regeneration of human endometrium is guaranteed by the proliferative capacity of endometrial mesenchymal stromal cells (E-MSCs). Due to this, the autologous infusion of E-MSCs has been proposed to support endometrial growth in a wide range of gynecological diseases. We aimed to compare two different endometrial sampling methods, surgical curettage and vacuum aspiration biopsy random assay (VABRA), and to validate a novel xeno-free method to culture human E-MSCs. Six E-MSCs cell samples were isolated after mechanical tissue homogenization and cultured using human platelet lysate. E-MSCs were characterized for the colony formation capacity, proliferative potential, and multilineage differentiation. The expression of mesenchymal and stemness markers were tested by FACS analysis and real-time PCR, respectively. Chromosomal alterations were evaluated by karyotype analysis, whereas tumorigenic capacity and invasiveness were tested by soft agar assay. Both endometrial sampling techniques allowed efficient isolation and expansion of E-MSCs using a xeno-free method, preserving their mesenchymal and stemness phenotype, proliferative potential, and limited multi-lineage differentiation ability during the culture. No chromosomal alterations and invasive/tumorigenic capacity were observed. Herein, we report the first evidence of efficient E-MSCs isolation and culture in Good Manufacturing Practice compliance conditions, suggesting VABRA endometrial sampling as alternative to surgical curettage.

## 1. Introduction

The human endometrium is characterized by a high proliferative potential, as it undergoes approximately 450 regenerative cycles during woman’s reproductive lifespan. Some studies reported the presence, in the basalis layer, of a small proportion of endometrial stromal cells, which were shown to be physiologically involved in the cyclic endometrial regeneration after menstrual loss [1,2,3,4,5]. In particular, endometrial mesenchymal stromal cells (E-MSCs) are clonogenic mesenchymal-like cells [4] localized in the perivascular space of endometrial small vessels [6] and able to express pericyte markers, such as SUSD2 [7,8]. E-MSCs were previously isolated by our group, both from the healthy endometrium and from peritoneal, pelvic endometriosis; when cultured, they displayed the ability to form plastic-adherent colonies with high proliferative potential, as well as the property of undergoing multi-lineage differentiation into osteoblasts, chondrocytes, adipocytes, or endothelial cells in response to specific culture conditions [9,10]. Due to their capacity of multi-lineage differentiation and their immunosuppressive properties, E-MSCs are considered suitable candidates for performing stem cell therapy [11,12]. It has been reported, in fact, that E-MSCs are capable to differentiate in vitro into endometrial epithelial and stromal cells when exposed to estradiol-containing media [13]. In addition, some studies have suggested the ability of human E-MSCs to repair endometrial damage both in animal models and in patients with Asherman’s Syndrome (AS), a pathological condition characterized by extensive endometrial disruption and intrauterine adhesions leading to hardly reversible infertility [14]. Stem cell therapy has been tested as a potential cure for severe AS. Human CD133+ bone marrow-derived stem cells (BMDSCs) were injected in a murine model of AS, and were able to induce the proliferation of endometrial vascular cells [15]. In humans, autologous CD133+ BMDSCs infusion, in conjunction with estrogenic replacement therapy, obtained enhanced endometrial angiogenesis and growth, increased volume and duration of menses, and a significant reduction of intrauterine adhesion score in patients with severe AS [16]. Stem cells for therapeutical use could also be obtained from the endometrium. Tan et al. [17] infused autologous menstrual blood-derived endometrial stromal cells (menSCs) to patients with AS, obtaining a significant increase in endometrial thickness that facilitated pregnancy, both spontaneous or after in vitro fertilization (IVF). More recently, E-MSCs spheroids transplanted in the uterus of rats with induced AS were able to restore fertility, allowing spontaneous pregnancies in which the litter size was higher than in control AS-affected rats receiving autologous bone marrow cells [18]. Taken together, these data suggest that cellular therapy with E-MSCs, might represent a promising option to treat severe endometrial defects causing infertility. Moreover, the endometrial tissue collection is considered a noninvasive method for obtaining E-MSCs, and more acceptable for patients with infertility than bone marrow collection. In addition, E-MSCs show a greater multiplication ability, and younger women show a higher multiplication ability [19]. In IVF, it could also be used to sustain endometrial growth in patients with recurrent implantation failure and scarce endometrial responsiveness to estrogens. For clinical use, however, E-MSCs are considered as advanced therapy medicinal products (ATMP), and, as a consequence, must be produced in compliance with Good Manufacturing Practice (GMP) rules [20], defining the highest standards of sterility, quality control, and documentation to ensure that cultured cells are safe. In this study, our aim was to isolate and expand human E-MSCs in GMP-compliant culture using the method that was previously set up by our group for human bone marrow-derived MSCs [21]. The main characteristic of this culture procedure was that it employs inactivated human platelet lysate (HPL) instead of fetal bovine serum (FBS), being free of animal-derived constituents. GMP guidelines must be followed in order to guarantee the safety of cellular products and their production and control at the quality standards required for their use (from the collection to the release). Some studies demonstrated that, although human MSCs are not highly immunogenic, when they are expanded in medium with FBS, an immune response was shown in some patients [22,23]. A single preparation of 10^8^ human MSCs expanded in standard condition, consisting of the use of FBS as additive in the culture medium, brings with it approximately 7–30 mg of FBS proteins [24]. Furthermore, animal derivatives could be a vehicle for animal pathogens and transmit infectious agents [25,26]. Thus, GMP guidelines aim to minimize use of FBS, preferring safer media supplementation alternatives [27]. Following these criteria, the use of HPL make the procedure compliant to the guidelines that must be followed during cell production for clinical use, as the animal component (FBS) in the culture medium is eliminated. We previously demonstrated that HPL is a more advantageous additive than FBS for MSC isolation and expansion from bone marrow [21], and our aim was to confirm the same efficacy in the culture of E-MSCs. Moreover, we also wanted to compare the effectiveness of two different methods of endometrial sampling to obtain E-MSCs: the surgical extensive curettage in general anesthesia (the E-MSCs obtained with this method will be defined as Cur-E-MSCs) and the office-based mini-invasive vacuum aspiration biopsy random assay, VABRA (the E-MSCs obtained with this method will be defined as Vab-E-MSCs). 

## 2. Results

### 2.1. Isolation of E-MSCs

**Figure 1:**
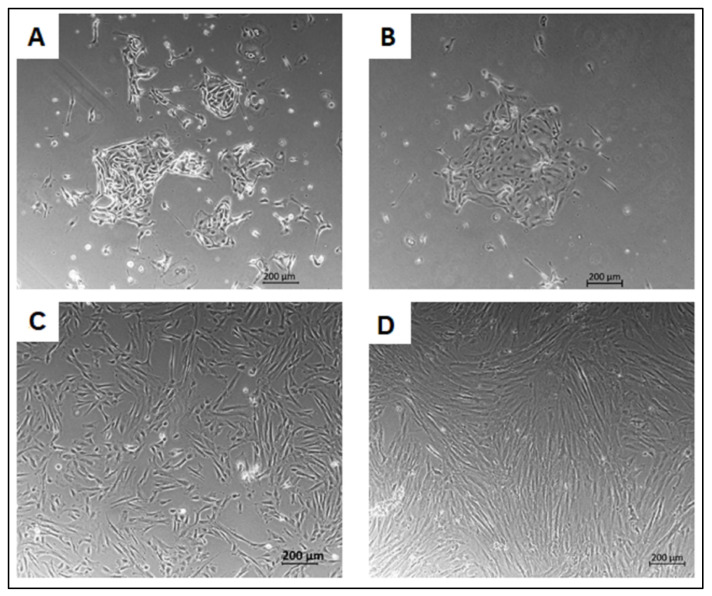
Morphology of E-MSCs obtained from curette and VABRA biopsies. Representative phase images at ×5 magnification of CFU-Fs after 4 days from seeding and of sub-confluent cell culture during the expansion at P5, respectively, in Cur-E-MSCs (**A**,**C**) and Vab-E-MSCs (**B**,**D**).

Six patients with mean age 29.6 ± 7.3 years (range 22–35) were enrolled; three of them underwent endometrial sampling by mechanical scraping using a surgical curette in the operating theatre during laparoscopy, and the other three underwent VABRA procedure consisting of random sampling of the endometrium using a plastic scraper and an aspiration pump in office during hysteroscopy (Supplementary Table S1). Adherent cells were observed in all cases after 7 days of culture, and in the following 15 days (first passage), a confluent layer formed of cells with an elongated, fibroblastic shape was rapidly generated. No morphological differences in the colony shape (Figure 1A,B) and during the expansion (Figure 1C,D) were observed comparing Cur-E-MSC and Vab-E-MSC.

### 2.2. Colony Formation and Cellular Expansion Analysis 

**Figure 2:**
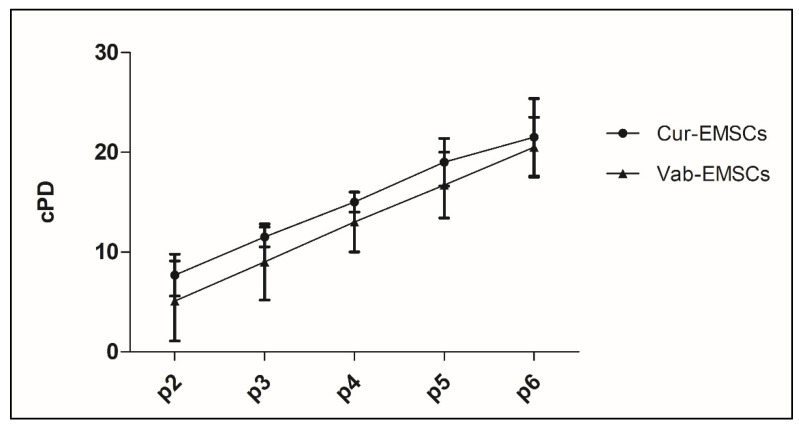
Growth kinetic curves of Cur-E-MSCs and Vab-E-MSCs during the expansion. Cellular growth is expressed as mean number of cumulative PD as a function of time, from the first to the sixth passage (p1–p6). The two populations showed no significant differences.

At each passage, E-MSCs showed a viability of 98–100% in all the analyzed samples, with no differences between Cur-E-MSC and Vab-E-MSC. The CFU-F number was calculated in relation to the initial cell number. Cur-E-MSCs showed a mean of 150 CFU-F per 10^6^ cells; and Vab-E-MSC, a mean of 126.2 CFU-F per 10^6^ cells, not significantly different. The cell proliferative capacity during expansion was expressed as population doubling (PD) using the following formula: Log_10_ N/Log_10_ 2, where N was the cell number of the detached cells divided by the initial number of seeded cells. The cellular expansion growth was expressed as cumulative PD (cPD, the sum of the PD of the current passage plus the PD of the previous ones). The cumulative PD after three passages was 11.5 ± 1.0 for the Cur-E-MSCs and 9.0 ± 3.8 for the Vab-E-MSCs, whereas after six passages it was 21.5 ±3.9 and 20.5 ± 3.0, respectively, showing no significant difference (Figure 2). 

### 2.3. Cytofluorimetric Analysis 

**Figure 3:**
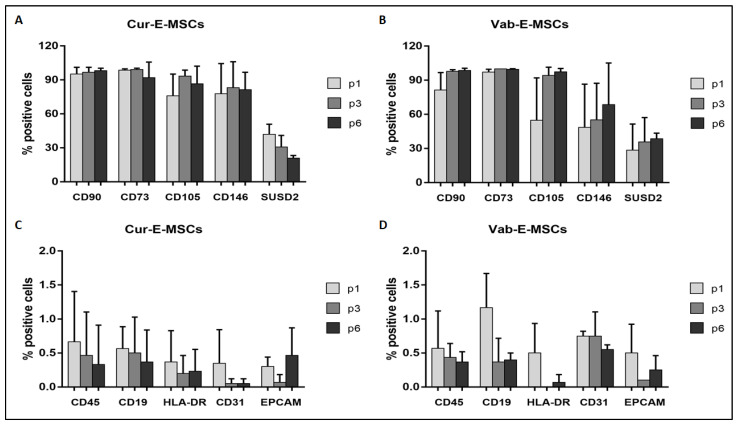
Flow cytometry analysis of mesenchymal, hematopoietic, and epithelial markers of Cur-E-MSCs and Vab-E-MSCs at the first, third, and sixth passage. Histograms show marker expression in Cur-E-MSCs (**A**,**C**) and Vab-E-MSCs (**B**,**D**) during the expansion at the first (p1), third (p3), and sixth (p6) cell passage. No differences of immunophenotype were observed during the expansion.

At each passage, cells were analyzed for their expression of mesenchymal, hemopoietic, and endothelial markers. Overall, Cur-E-MSCs and Vab-E-MSCs expressed comparable levels of all tested markers (Table 1). The presence of epithelial, endothelial, and hematopoietic cell contamination was excluded by the absence of EPCAM, CD31, CD45, CD19, and HLA-DR (Figure 3). No significant difference was observed in the marker expression at the first, third, and sixth cell passage between Cur-E-MSCs (Figure 3A,C) and Vab-E-MSC (Figure 3B,D). These data indicate that the two E-MSC populations had a similar mesenchymal phenotype, steadily maintained during in vitro culture.

### 2.4. Stemness Evaluation

**Figure 4:**
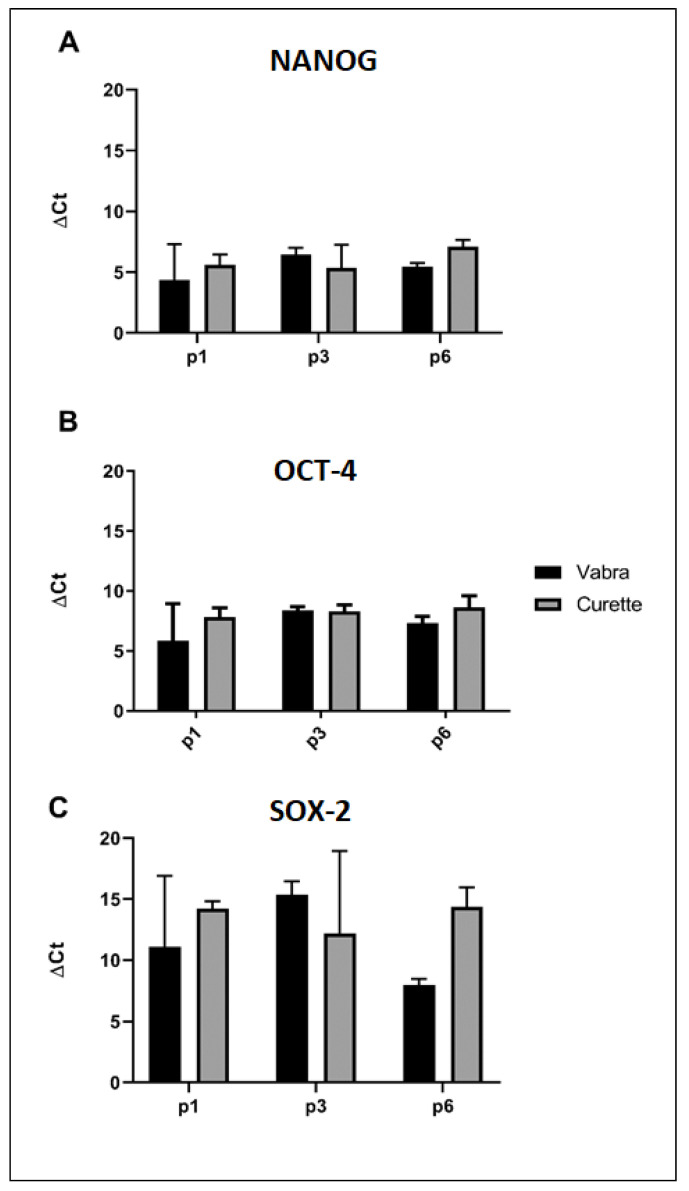
mRNA expression of NANOG, OCT4, and SOX2 in Cur-E-MSCs and Vab-E-MSCs during the expansion. Mean expression of NANOG (**A**), OCT4 (**B**), and SOX2 (**C**) in Cur-E-MSCs and Vab-E-MSCs at the first (p1), third (p3), and sixth (p6) cell passage. Data were expressed as ΔCt values, calculated by CT target gene – CT housekeeping gene, during the expansion.

The mRNA expression of the stemness-related genes, Homeobox protein (NANOG), octamer-binding transcription factor 4 (OCT4), and SRY (sex determining region Y)-box 2 (SOX2) were analyzed by real time PCR. No significant differences between Cur-E-MSCs and Vab-E-MSCs were observed in the overall mean gene expression (Figure 4A) and in gene expression at different cell passages (Figure 4B,C), showing that both cell populations displayed similar stemness phenotype, steadily maintained during the in vitro culture. 

### 2.5. In Vitro Differentiation

**Figure 5:**
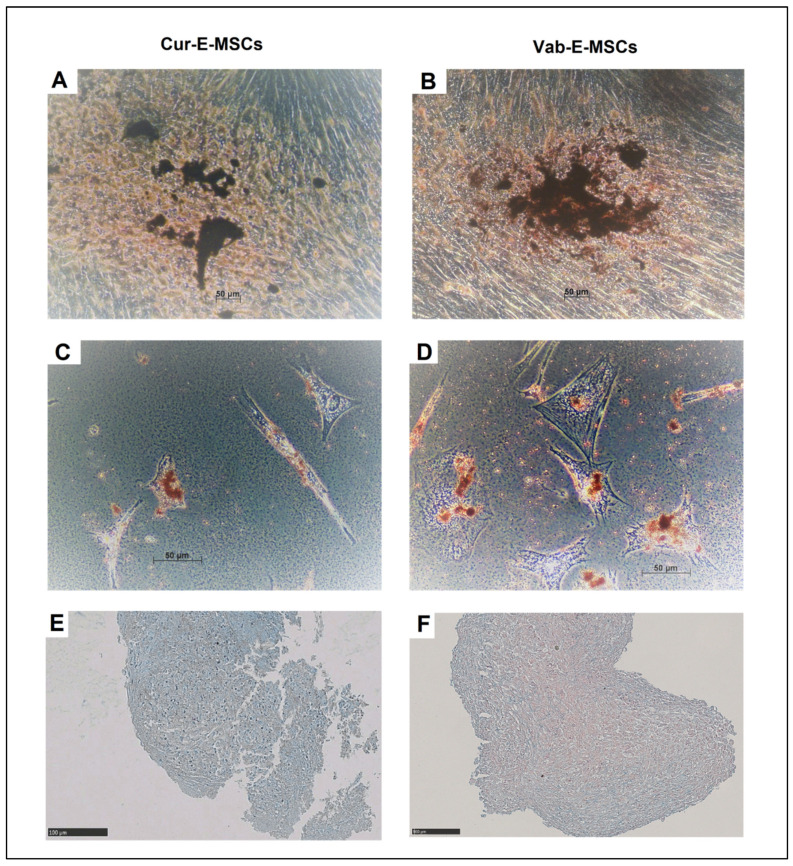
In vitro differentiation potential assay after 3 weeks of specific induction (three different conditions). Induced differentiation in Cur-E-MSCs (left column), and Vab-E-MSCs (right column). Von Kossa staining (**A**,**B**) shows the presence of calcium oxalates, indicating osteogenic differentiation. Oil red O stain (**C**,**D**) shows intra-cytoplasmatic vacuoles, indicating differentiation into adipocytes. Alcian blue stain (**E**,**F**), shows the hyaluronic acid content, typical of chondrocytes. Magnification: 20×.

At the third passage, cells of both studied lines were induced to differentiate into different lineages using specific media. After osteogenic differentiation, around 60% of cells of both lines contained crystals of calcium ossalate (Figure 5A,B). Both cell lines showed morphological changes and the generation of adipocytes was found in less than 5% of the viable population, revealed as round cells accumulating little intracytoplasmic lipid vacuoles, positive at Oil Red O staining (Figure 5C,D). At a comparable level, chondrocyte differentiation was achieved in a reduced fraction of cell population, appearing as cells aggregated in spheres positive to Alcian Blue stain of hyaluronic acid and sialomucin (Figure 5D,E). Overall, both Cur-E-MSCs and Vab-E-MSCs showed comparable multilineage differentiation in appropriate culture conditions.

### 2.6. Karyotype Analysis

**Figure 6:**
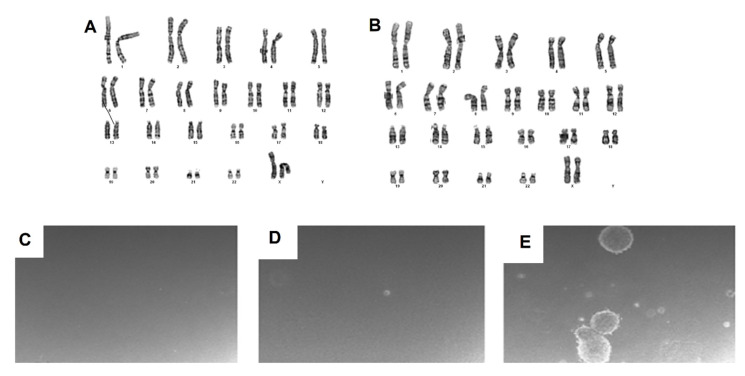
Karyotype analysis and soft agar assay to assess genetic modification and/or tumorigenesis potential in Cur-E-MSCs and in Vab-E-MSCs. Normal karyotype at the sixth passage (**A**,**B**), showing no genetic alteration during the expansion of either Cur-E-MSCs (**left**) and Vab-E-MSCs (**right**). No colonies were observed in either Cur-E-MSCs or Vab-E-MSCs in soft agar assay (**C**,**D**), or in (**E**) primary osteosarcoma cells used as positive control.

Cur-E-MSCs and Vab-E-MSCs were analyzed at the first, third, and sixth passage. All karyotypes were reported as normal (46, XX) and no chromosomal alterations (translocations, deletion, additions, or aneuploidies) were noticed, showing no detrimental effect of in vitro culture on chromosomal stability (Figure 6A,B).

### 2.7. Invasion Assay

Both Cur-E-MSCs and Vab-E-MSCs did not show invasive capacity and tumorigenic activity in the soft agar culture assay (Figure 6C,D); differently, the Sjsa positive control (osteosarcoma cell line) clearly formed colonies after 21 days in the same conditions (Figure 6E).

## 3. Discussion

According to cell therapy regulation, E-MSCs must be isolated and produced in GMP conditions, using standardized techniques for their clinical use. In the present study, we aimed to validate a xeno-free method to isolate and expand E-MSCs, which we previously used to validate human bone marrow-derived MSC manufacturing in GMP conditions [21,27]. In order to avoid digestive enzymes and FBS, we set up the isolation procedure using mechanical digestion to homogenate the endometrial samples. Then, we cultured the cells in presence of HPL instead of FBS, obtaining a medium devoid of animal components. We showed, herein, that our method could be successfully applied to isolate and expand human E-MSCs. In fact, our E-MSCs showed remarkable adherent properties, colony formation, and proliferative potential, as well as mesenchymal stromal characteristics matching the criteria of the International Society for Cellular Stem Cells International Therapy (ISCT) [28,29]. The cultured E-MSCs showed stemness characteristics, as confirmed by the expression of NANOG, OCT4, and SOX2, and mesenchymal phenotype, that were steadily maintained during the in vitro culture up to the sixth passages. E-MSCs underwent adipocyte and chondrocyte differentiation in a limited fraction of cells, whereas higher percentage was able to differentiate into osteoblasts. These results are in line with previous studies, reporting that only a small fraction of E-MSCs exhibited trilineage differentiation. In particular, in previous studies, it appears that the differentiation potential was observed only in a small percentage of cells after a sorting process: ≈1.5% of CD146+PDGFR-β+ cell [6], ≈4% of SUSD2+ cells [30], ≈1.7% in the epithelium, and 0.4% of stromal fraction of endometrial side population (SP) cells [31]. Although a cell sorting was not performed in the current study, our data confirmed previous observations, as the heterogeneous overall population deriving from fresh biopsies was cultured. Interestingly, E-MSC expressed stem cell marker during the expansion also after the third passage, and no chromosomal alterations were observed in all expanded batches, suggesting no detrimental effects of the prolonged culture on stemness and chromosomal stability. Finally, they did not show invasive and growth capacity in soft agar, confirming the complete safety of this population for clinical use. Overall, quality controls needed to guarantee the safety for clinical administration of these E-MCSs to patients resulted compliant at the batch release as required by the GMP regulations [20,26,32], supporting their potential clinical use to enhance endometrial growth. We also compared the efficiency of two endometrial sampling methods: surgical curettage, previously used by our group to get MSCs from the endometrium and from both ovarian and peritoneal endometriosis [10]; and the vacuum aspiration biopsy random assay (VABRA), a less invasive, cheap office-based method not requiring anesthesia. Herein, we showed that isolation and expansion of E-MSCs were equally efficient, starting from samples obtained by either biopsy method, and that the cell characteristics were not influenced by the endometrial sampling technique. The total amount of cells recovered and obtained after three passages of in vitro culture was comparable to those observed previously by our group [9,10]. Notably, VABRA allowed the recovery of a reduced amount of endometrial tissue compared to the surgical curettage, requiring a prolonged in vitro culture in the very first cell passage to obtain an adequate cell population for further experiments. However, as previously described, the proliferation rate in the subsequent cell passages was compared between the two populations. In addition, VABRA sampling was performed in patients with suspected endometrial polyps, requiring further laboratory precautions, such as strictly selective culture media and an accurate karyotype evaluation, in order to exclude the pivotal contamination of cancer cells. The limited sample size could represent a weakness of the current study, but following Guidelines for Good Manufacturing Practice, Annex 15, about qualification and validation [33], we considered three batches for each condition acceptable for our study. However, the data obtained in this study highlighted the equivalence of the in vitro properties of the E-MSCs isolated, comparing the mechanical curettage to the random aspiration and opening the possibility for further clinical use. We have enrolled patients with endometrial polyps, because healthy women cannot undergo hysteroscopy and VABRA for ethical issues.

It is rather well established that when endometrial thickness does not reach a peak value of 7 mm in the ovulatory phase of the cycle, the occurrence of pregnancy, either spontaneous or after transfer of an in vitro-produced embryo, is significantly less likely [34,35]. When endometrial growth is inadequate, estrogen administration during the menstrual cycle is frequently the chosen treatment, yet this therapeutic approach is efficient only in a limited number of patients due to the rather frequently observed limited responsiveness of the tissue to ovarian hormones [36]. The extreme form of endometrial impairment is Asherman’s syndrome (AS) [14]. Current treatments for AS include hysteroscopic adhesiolysis, followed by antibiotic and hormonal therapy aimed at avoiding recurrent adhesive disease; however, they are poorly effective in restoring endometrial growth and function [37]. As epithelial and stromal cells with stem cell characteristics are found in the basalis layer of the human endometrium, where they were shown to be involved in the cyclic endometrial regeneration [1,2,3,4], it was hypothesized that a thin endometrium which is refractory to estrogens could be the result of a defective number or function of endometrial stromal cells. Following this idea, the infusion of autologous E-MSCs could represent a novel therapy for endometrial regeneration and growth enhancement [38,39], and the VABRA method of harvesting the E-MSCs makes the use of these cells in clinical trials less invasive and more appropriate for patients. In fact, it was demonstrated that they highly express Cysteine angiogenic inducer 61 (CYR61) that plays an important role in the induction of neoangiogenesis in the damaged endometrium [40]. In addition, extracellular vesicles collected from E-MSCs participate in adaptive/innate immune responses and complement activation, antigen processing/presentation, and negative regulation of apoptosis, which can be used as immunomodulatory regulators for the treatment of inflammation-related diseases [41]. As previously written, E-MSCs are capable to differentiate in vitro into endometrial epithelial and stromal cells when exposed to estradiol-containing media [13]. Therefore, E-MSCs spheroids transplanted rat uterus with induced AS were able to restore fertility, allowing spontaneous pregnancies in which the litter size was higher than in control AS-affected rats receiving autologous bone marrow cells [18].

For these reasons, it was more efficient to use E-MSCs instead bone marrow-derived MSCs for clinical trials in endometrial diseases.

## 4. Materials and Methods

### 4.1. Patients

E-MSCs were taken from healthy endometrial samples obtained from six patients enrolled at the Department of Surgical Sciences, University of Torino, between May 2018 and June 2019. The study was conducted after approval by the Ethics Review Board (Prot. N° 0055438, 28 May 2018) and preoperative written informed consent was obtained from each patient. We enrolled patients with normal cavity evaluations with suspected endometrial polyps, which were ultimately not present on hysteroscopic evaluation or benign ovarian cyst, as frequent candidates to endometrial sampling with VABRA or surgical operation, respectively. Endometrial sampling was performed either by surgical curettage from patients operated for benign ovarian cysts or hydrosalpinx in general anesthesia, or by the VABRA procedure, performed during diagnostic hysteroscopy, without the need of any anesthesia [34]. 

### 4.2. E-MSC Isolation and Culture

The obtained E-MSCs were distinguished, according to the sampling procedure, as Cur-E-MSCs (curettage-derived) and Vab-E-MSCs (VABRA-derived). Endometrial samples were collected in a sterile tube containing α-Mem (Sigma Aldrich, St. Louis) with 1% L-glutamine (Gibco, USA), 1% penicillin/streptomycin (Sigma Aldrich), and 10% HPL (Human Platelet Lysate), prepared by the ISO certified/accredited center “Production and Emocomponent Validation Centre” of Turin, as previously described [21], using a standardized operative procedure. The standardization of the product was made possible by the use of 60 blood donors and the pool of ten Buffy-Coat derived platelet concentrate (PC) units and adding heparin (200 U/mL) to avoid gelation of the medium in culture. Quality parameters of each prepared PC was complied with Italian and European guidelines and regulatory requirements; platelet content per final unit: ≥2 × 1011; residual leucocyte content: <1 × 106 per final unit.

Cells were seeded at a density of 10,000 cells/cm^2^ in the same medium of the collection in six-well multiwell plates for CFU evaluations, and in T75 culture flasks (d). After 7 days, detached cells were discarded, whereas the adherent cells were refed every 2–3 days. After reaching confluence, which was achieved usually 10 days after seeding, cells were detached using Trypsin (Sigma Aldrich) and replated at a density of 1000 cells/cm^2^ for six passages as maximum. A sterility test (Bact Alert; Biomerieux) was performed on the endometrial samples before seeding in order to exclude bacterial or fungal contamination. At each passage, the cultured cells were analyzed for growth, viability, immunophenotype, RNA expression of stem cells markers, and chromosomal stability. The differentiative potential and invasion assay were evaluated at the third culture passage. 

### 4.3. Colony Formation Assay, Cell Viability and Proliferative Capacity

To evaluate the clonogenic potential, fibroblastic colony forming unit (CFU-F) assay was performed for each cell batch. After homogenization, cells were seeded in duplicate in six-well plates at a density of 10,000; after 7 days from seeding, they were fixed in acetone/methanol (1:1) and stained with the May Grunwald Giemsa dye (Sigma Aldrich) to allow cell count. Cell count and viability assays of the cells before seeding were performed using Burker chamber after treatment with Turk’s liquid to lyse the red blood cells and 1:1 Trypan Blue staining. Clusters including more than 50 cells were considered colonies. The CFU-F value was calculated as mean of the number of colonies obtained after seeding and expressed as number of CFU-F frequency in 10^6^ seeded cells. The cell proliferative capacity during expansion was expressed as population doubling (PD) using the following formula: Log10N/Log102, where N was the cell number of the detached cells divided by the initial number of seeded cells. The cellular expansion growth was expressed as cumulative PD (cPD), as previously described [35].

### 4.4. Flow Cytometric Analysis

Cell suspension (1–2 × 10^6^) was incubated with antibodies for 20 min at 4 °C in 100 µL of phosphate buffered saline (PBS). The following anti-human monoclonal antibodies, all fluorescein isothiocyanate (FITC)- or phycoerythrin (PE)- or allophycocyanin (APC)-conjugated, were used at 1:10 dilution: anti-CD90 FITC (ref: IM1839U), anti-CD73 PE (ref: B68176), anti-CD105 PC7 (ref: B43293), anti-CD45 FITC (ref: IM0647), anti-CD34 FITC (ref: IM1870), anti-CD14 FITC (ref: B36297), anti-HLA-DR PE (ref: IM1639), anti-CD19 APC (ref: IM2470), anti-CD31 FITC (ref: IM1431U) (Beckman Coulter, Brea, CA, USA), anti-CD146 APC (clone: REA773), anti-SUSD2 APC (clone: W5C5), and anti-EPCAM FITC (clone: HEA-125) (Miltenyi Biotech, Bergisch Gladbach, Germany). As negative control, cells were incubated without antibodies. Labelled cells were washed with PBS and analyzed using Navios cytometer (Beckman Coulter, CA, USA).

### 4.5. Real-Time PCR Analysis

Total RNA extraction was performed using the Maxwell automatic extractor (Promega, Madison, WI, USA) according to the manufacturer’s protocol, and the obtained RNA was quantified by Nanodrop (Thermo Scientific, Wilmington, DE, USA). Single strand cDNA was produced from 1 μg of total RNA using SuperScript™ II Reverse Transcriptase (Invitrogen-Life Technologies Corp, Carlsbad, CA, USA) and the GeneAmp 9700 Thermal Cycle (Applied Biosystems, Foster City, CA, USA). Real-time PCR experiments were performed in a 20 µL reaction mixture containing 100 ng cDNA template and primers designed by Primer ExpressTM version 3.0 (Applied Biosystems, Foster City, CA, USA). Glyceraldehyde-3-phosphate dehydrogenase (GAPDH) mRNA was used to normalize RNA inputs. Gene expression was performed for the following markers: glyceraldehyde 3-phosphate dehydrogenase (GAPDH), 5′-CAAGGTCATCCATGACAAC-3′, 5′-GTGGCAGTGATGGCATGGAC-3′; Homeobox protein (NANOG), 5′-GCCAGGGGTCTCGATCTC-3′, 5′-GGTGGCTCACGCCTGTAAAT-3′; octamer-binding transcription factor 4 (OCT4), 5′-ACCCACACTGCAGCAGATCA-3′, 5′-CACACTCGGACCACATCCTTCT-3′; SRY (sex determining region Y)-box 2 (SOX2), 5′-TGCGAGCGCTGCACAT-3′, 5′-GCAGCGTGTACTTATCCTTCTT-3′. A respective amount of 500 nmol of specific primers and 200 nmol of specific probes were used. Relative quantification of the products was performed using a 96-wells plate with the Taqman enzyme amplification process (ABI PRISM 7500 real time system, Life technologies, Austin, TX, USA). Thermal cycling conditions were as follows: activation of GoTaq^®^ qPCR Master Mix (Promega) at 95 °C for 2 min, followed by 40 cycles of amplification at 95 °C for 15 s and 60 °C for 1 min. We compared the expression of target gene in the different batches using ΔCt values calculated by CT target gene – CT housekeeping gene during the expansion.

### 4.6. Karyotype Analysis

Karyotype analysis was performed after cells were arrested at metaphase by incubation with Colcemid (Invitrogen Corporation, Grand Island, NY, USA), maintained in a hypotonic solution (0.075 M KCl), fixed with methanol/acetic acid 3:1 (Merck, Milan, Italy), and stained with Giemsa using standard laboratory protocols. At least 20 metaphases were analyzed using MackType software (Nikon Corporation, Tokyo, Japan) according to the International System for Human Cytogenetic Nomenclature.

### 4.7. Differentiation Potential Assay

For differentiation experiments, E-MSCs were cultured at the third passage in osteogenic, adipogenic, and chondrogenic mediums, according to the manufacturer’s instructions. Briefly, for osteogenic induction, 45,000 cells were plated in each well of six well plates and cultivated in StemMACS OsteoDiff Media (Miltenyi, Bergisch Gladbach, Germany). After 21 days, osteogenic differentiation was demonstrated by the accumulation of calcium (crystalline hydroxyapatite detection by Von Kossa staining). For adipogenic differentiation, 75,000 cells were cultured in StemMACS AdipoDiff Media differentiation medium (Miltenyi, Bergisch Gladbach, Germany) for 21 days, after which the presence of intracellular lipid vesicles was assessed after fixation with paraformaldehyde vapors and Oil Red O staining. For chondrogenic differentiation, an aliquot of 250,000 cells were cultured in StemMACS ChondroDiff Media differentiation medium (Miltenyi, Bergisch Gladbach, Germany) for 21 days in 15 mL polypropylene culture tubes. During chondrogenic differentiation, cellular growth occurred as cellular aggregates floated freely in suspension. The pellet was included in paraffin and stained with Alcian Blue to identify the presence of hyaluronic acid and sialomucin.

### 4.8. Soft Agar Assay

To exclude the tumorigenic capacity and invasiveness of E-MSCs, the soft agar assay was performed using a commercial tumor mesenchymal cell line (Sjsa) as a positive control. A 4% solution of noble agar (Becton, Dickson and Company, Le Port de Claix, France) was prepared, as previously described in The Soft Agar Colony Formation Assay [36]. The E-MSCs at P3 in each culture condition were harvested, washed, and seeded at a final density of 5000 cells/well in 6-well plates in duplicate. The test was performed with the use of 0.8% and 0.4% agar in Alpha Mem added with 1% of L-Glutammine, 1% of Pen/Strept, and 10% of HPL, arranged at the base and on the surface, respectively. The cells were incubated for 21 days at 37 °C in the presence of 5% CO_2_, and 100 µL of culture medium was added twice a week. After incubation, the colonies were counted with the use of the inverted microscope.

### 4.9. Statistical Analysis

Results were analyzed by GraphPad Prism V7 software (GraphPad Software Inc., San Diego, CA, USA). Data were analyzed for normality using the Shapiro–Wilk test, and then expressed as mean ± SD. Differences in mRNA expression level and in the immunophenotype among groups were investigated using 2-way Anova with Bonferroni’s multiple comparison test. Significance was set at *p* < 0.05. 

## 5. Conclusions

In conclusion, herein we report the validation of a new GMP-compliant method to isolate E-MSCs from human endometrium, as well as the comparison between cells obtained by two different sampling techniques, surgical curettage and VABRA. The novel culture method proposed is characterized by a mechanical digestion (instead of enzymatic digestion) of the biopsied sample and by the use of inactivated HPL (instead of FBS) that avoids the presence of animal-derived products, thus preventing the risk of transmitting infectious agents and of eliciting immunization. The isolated and expanded E-MSCs by this culture method showed all the characteristics of MSCs as defined by the ISCT, preserving the stemness markers and showing no karyotype modification or tumorigenic potential. Our observations suggest that (a) the novel culture technique proposed herein guarantees a safe and efficient GMP-compliant method to isolate E-MSC for future clinical application, and (b) that a simpler, cheaper, and quicker endometrial sampling technique like VABRA can be successfully used to obtain E-MSCs instead of the surgical curettage in general anesthesia.

## Figures and Tables

**Table 1:** Cytofluorimetric evaluation of surface marker expression of Cur-E-MSCs and Vab-E-MSCs. Quantitative mean expression of mesenchymal, hematopoietic, and endothelial markers was assessed by FACS analysis. Values represent the percentage of positive cells and are expressed as mean ± SD. The immunophenotype of both E-MSC populations showed no significant difference during the culture.

Marker	Cur-E-MSCs (*n* = 3) (%)	Vab-E-MSCs (*n* = 3) (%)	*p* Value
CD90	97.2 ± 2.7	93.2 ± 7.9	ns
CD73	97.5 ± 4.7	99.2 ± 0.8	ns
CD105	82.2 ± 20.5	70.3 ± 27.3	ns
CD146	80.6 ± 27.6	62.8 ± 20.2	ns
SUSD2	30.5 ± 9.8	32.0 ± 24.7	ns
CD45	0.6 ± 0.5	2.2 ± 0.7	ns
CD19	0.6 ± 0.2	0.7 ± 0.4	ns
HLA-DR	0.3 ± 0.3	0.3 ± 0.4	ns
CD31	2.2 ± 2.5	2.8 ± 2.0	ns
EPCAM	0.3 ± 0.3	0.5 ± 0.4	ns

## Data Availability

All data generated or analyzed during this study are included in this published article. The data obtained in this study are available from the corresponding author upon request.

## Supplementary Materials

The following supporting information can be downloaded at: https://www.mdpi.com/article/10.3390/ijms23041931/s1.

## Abbreviations

AS	Asherman syndrome
APC	Allophycocyanin
ATMP	Advanced therapy medicinal products
BMDSCs	Bone marrow-derived stem cells
CD	Cluster of differentiation
CFU-F	Colony forming units-fibroblast
Cur-E-MSCs	Endometrial mesenchymal stromal cells isolated by curette
E-MSCs	Endometrial mesenchymal stromal cells
EPCAM	Epithelial cell adhesion molecule
FACS	Fluorescence-activated cell sorting
FITC	Fluorescein isothiocyanate
FBS	Fetal bovine serum
GMP	Good Manufacturing Practice
HLA-DR	Human leukocyte antigen–DR isotype
HPL	Human platelet lysate
ISCT	International Society for Cellular Stem Cells International Therapy
IVF	In vitro fertilization
MenSCs	Menstrual blood-derived endometrial stromal cells
NANOG	Nanog homeobox
OCT4	Octamer-binding transcription factor 4
PE	Phycoerythrin
PCR	Polymerase chain reaction
PD	Population doubling
SOX2	SRY (sex determining region Y)-box 2
SRY	Sex Determining region Y
SUSD2 (W5C5	Sushi domain containing 2
Vab-E-MSCs	Endometrial mesenchymal stromal cells isolated by VABRA
VABRA	Vacuum aspiration biopsy random assay
