# Supplementary material for: A Novel Xeno-Free Method to Isolate Human Endometrial Mesenchymal Stromal Cells (E-MSCs) in Good Manufacturing Practice (GMP) Conditions
Source: Int J Mol Sci. 2022 Feb 9;23(4):1931. doi: 10.3390/ijms23041931 (PMC8876308; doi:10.3390/ijms23041931)
Supplement: Supplementary file 1 [file ijms-23-01931-s001.zip › ijms-1563546-supplementary.pdf]

Table S1. Clinical characteristics of patients enrolled for the study.

Curette					
**Sample**	**Age**	**BMI**	**Gravidity/Parity**	**Indication**	**Other Pathologies**
E-MSC01	22	23.6	0/0	Benign ovarian cyst	none
E-MSC05	19	22.3	1/0	Benign ovarian cyst	none
E-MSC09	33	21.8	1/1	Benign ovarian cyst	none
**VABRA**					
**Sample**	**Age**	**BMI**	**Gravidity/Parity**	**Indication**	**Other Pathologies**
E-MSC02	35	24.7	1/1	Endometrial polyp	none
E-MSC04	33	20.4	0/0	Endometrial polyp	none
E-MSC07	35	21.5	1/1	Endometrial polyp	none
